# Circulating microRNA in patients with popliteal and multiple artery aneurysms

**DOI:** 10.1016/j.jvssci.2021.04.003

**Published:** 2021-05-15

**Authors:** Dick Wågsäter, Hans Ravn, Anders Wanhainen, Helena Isaksson, Martin Björck

**Affiliations:** a Department of Medical Cell Biology, Uppsala University, Uppsala, Sweden; b Department of Surgical Sciences, Section of Vascular Surgery, Uppsala University, Uppsala, Sweden; c Department of Vascular Surgery, Hospital Lillebaelt, University of Southern Denmark, Kolding, Denmark; d Department of Surgical and Perioperative Sciences, Umeå University, Umeå, Sweden; e School of Health and Medical Sciences, Örebro University, Örebro, Sweden

**Keywords:** Genetic, Plasma, Marker, Aneurysm, Popliteal artery, Aorta, MicroRNA

## Abstract

**Background:**

Patients with popliteal artery aneurysm (PA) often have multiple aneurysms, such as bilateral disease or a concomitant abdominal aortic aneurysm (AAA). microRNAs (miRs) are regulators of biological processes and have been investigated as biomarkers for AAA. The aim of this study was to explore if the presence of multiple aneurysms and/or location correlated with miR levels in blood.

**Methods:**

Using quantitative polymerase chain reaction, 23 miRs were analyzed in plasma from 183 patients with PA.

**Results:**

Fifteen of the miRs were associated with the number and/or location of aneurysms (1.3- to 2.1-fold changes). Levels of miR-93 (1.4-fold) and miR-215 (1.6- to 1.9-fold) were changed in all compared groups. MiR-24 and miR-23a were altered in those with AAA (1.4- and 1.5-fold, respectively) or bilateral PA (1.5- and 1.4-fold, respectively), compared with in those without. MiR-145 were significantly altered (1.7-fold) in those with isolated PA and AAA, whereas miR-326 were altered in those with bilateral (2.3-fold) and isolated PA (1.9-fold).

**Conclusions:**

Different miRs seem to be important or to be markers for different subgroups of patients with PA. The identified miRs target vascular smooth muscle cell proliferation and vascular inflammation. Further studies are needed to increase the understanding of the pathogenesis of aneurysmal disease.

## Clinical Relevance

Patients with popliteal artery aneurysm often have multiple aneurysms, such as bilateral disease or concomitant abdominal aortic aneurysms, but the molecular pathogenesis of the disease is not fully understood. MicroRNAs are important regulators of gene expression and biological processes and have recently been investigated as possible biomarkers for abdominal aortic aneurysm. This study identified 11 microRNAs that were altered in subgroups of patients with popliteal artery aneurysm, which could be important regulators to study in interventional studies.

**Article Highlights**

• **Type of Research:** Human study
• **Key Findings:** Plasma from 183 patients with popliteal artery aneurysm were analyzed for 23 different microRNAs (miRs) with relevance to abdominal aortic aneurysm and compared with subgroups with concomitant abdominal aortic aneurysm, bilateral popliteal aneurysm and those with multiple aneurysm. Fifteen of the miRs showed differing levels between the study groups (1.3- to 2.1-fold changes).
• **Take Home Message:** miR that have previously been identified in abdominal aortic aneurysm are also altered among patients with popliteal artery aneurysm.

Popliteal artery aneurysm (PA) is a relatively rare aneurysmal disease affecting about 0.1% to 0.3% of the elderly male population; its molecular pathogenesis is not fully known. The disease is very rare among women, and only about 5% of those affected are female.^1^ However, among 65-year-old men with AAA detected through screening, the prevalence was recently reported to be 14.2%.^2^ Patients with PA often develop multiple aneurysms, such as an abdominal aortic aneurysm (AAA), which is a much more frequent aneurysmal disease; approximately 1.3% to 2.2% of 65-year-old men in northern Europe are affected.^3^^,^^4^ Rupture, which is the main life-threatening event affecting patients with AAA, is uncommon in patients with PA,^5^ who are instead most often affected by acute limb-threatening ischaemia.^6^

We recently screened AAA patients for the 172 most commonly expressed microRNAs (miRs) in plasma.^7^ Several miRs associated with AAA and aneurysm growth were identified, including genes involved in AAA disease, such as those encoding IL-1β, IL-6, interferon-γ, tumor necrosis factor, transforming growth facto-β, matrix metalloproteinase-2, matrix metalloproteinase-9, CXCL10, and collagen I, as well as novel genes and pathways. MiRs are short nucleotides that are stable in plasma and can regulate gene expression and biological processes through various mechanisms.^8^ Some miRs have specific targets, but most affect several hundred genes.

The aim of this study was to explore, in a population of patients with a high proportion of multiple aneurysms, if the presence of multiple aneurysms and/or location correlated with miR levels of miRs found in previous studies with relation to aneurysm disease.

## Methods

### Patient cohort

A total of 571 patients were initially identified in the Swedvasc registry as having undergone one or more PA operations between 1987 and 2002, on 717 legs in total. All these patients' medical records were analysed.^6^^,^^9^ Two-hundred forty of these patients consented to and participated in interviews. One hundred ninety patients participated in the reexamination, which was performed during 2006.^10^^,^^11^ Of them, seven had to be excluded owing to unsuccessful DNA extraction. Thus, a total of 183 patients were included in this analysis. Patient characteristics are described in Supplementary Tables I-III. The reexamination included ultrasound screening of the infrarenal aorta, the common iliac arteries, the common femoral arteries, and the bilateral popliteal artery segments. Aneurysm was defined as an aorta of greater than 30 mm, a common iliac artery of 20 mm or greater or 50% larger than the bilateral side, or a common femoral, and/or bilateral popliteal artery of 15 mm or higher. A total of 423 aneurysms were identified (range, 1-7; mean, 2.3 per patient). All the 183 index PAs had been operated on at the time of blood sampling. Of the 423 aneurysms, 299 (71%) had required surgery before blood sampling.

### Real-time quantitative polymerase chain reaction analysis of miRs

EDTA blood samples were centrifuged at 2000×*g* for 7 minutes within 3 hours from collection. Plasma was separated and frozen directly at –20 °C. After concluding investigations at each site, all samples were then shipped together on dry ice to the collecting biobank at Örebro University Hospital for storage at –80 °C. No sample was stored longer than 1 week at –20 °C.

RNA was extracted from 200 μL plasma from each of the 183 patients and reverse transcribed using the miRCURY LNA Universal RT microRNA polymerase chain reaction, polyadenylation, and cDNA synthesis kit (Exiqon, Vedbaek, Denmark) as previously described in greater detail.^7^

Amplification of the miRs (UniSp2 and UniSp6 (spike in controls), as well as miR-10b-5p, 21-5p, 23a-3p, 24-3p, 26a-5p, 33a-5p, 93-5p, 99a-5p, 103a-3p, 125a-5p, 136-5p, 145-5p, 152-3p, 192-5p, 194-5p, 205-5p, 215-5p, 221-3p, 222-3p, 326, 335-5p, 451a, and let-7i-5p were performed in a Roche Lightcycler 480. Reactions with amplification efficiency of less than 1.6 were removed. All data were normalized against miR-93 and miR-152, which were found to be the most stable miRs, to correct for potential overall differences between the samples.

### Statistical analysis

Independent sample *t* tests were used for comparing continuous variables, and the Fisher exact test for comparing dichotomous clinical variables, to test differences between two groups. All miR measurements are shown as average normalized quantitation cycle values for each group and fold changes with standard deviations. Two-group comparisons were performed using the Student *t* test. Adjustments of covariates were performed using binary logistic regression analysis. Correlation was tested with Pearson's coefficient. *P* values of less than .05 were considered statistically significant. Statistical analysis was performed with SPSS version 24.0 (SPSS, Inc, Cary, NC).

## Results

Of the 183 patients with PAs, 83 had a concomitant AAA (Supplementary Table I), 51 a concomitant iliac aneurysm, 51 a femoral artery aneurysm, and 105 a bilateral PA (Supplementary Table II); thus, a total of 423 different aneurysms were included. Of the patients, 56 had an isolated PA and 127 multiple aneurysms (defined as ≥2 aneurysms) (Supplementary Table III). Age was older among those with AAA, bilateral PA, and multiple aneurysms and was adjusted for in the statistical analysis. Hypertension was more frequent among those with multiple aneurysms and adjusted for in the analysis. Twenty-five miRs (including controls) were analyzed in plasma from 183 patients with PA. Nine miRs were identified in all samples, with an average of 21 miRs detectable per sample. In total, 15 of the miRs showed different levels in the different study groups of patients with PA.

### Expression of miRs in plasma in patients with PA with concomitant AAA

Eleven miR levels (23a, 24, 26a, 33a, 93, 103a, 125a, 145, 192, 215, and 451a) were changed (1.3-fold to 2.0-fold) in patients with PA with concomitant AAA, compared with in those without, with an average area under the curve (AUC) ranging from 0.58 to 0.65 at the highest (Table I). The differences for three of these miRs (23a, 33a, and 145) remained significant when adjusting for age differences, which was the covariate that differed between the groups. Only differences for miR-145 levels remained significant in a multiple regression analysis including all miRs in the equation. The AUC for miR-145 combined with age was slightly increased, from 0.65 to 0.68 (0.61-0.76).

**Table I:** MicroRNAs (*miRs*) in plasma that are different when comparing patients with a popliteal artery aneurysm (PA) with or without concomitant abdominal aortic aneurysm (*AAA*)

miR	Mean dCq ± SD no AAA (n = 100)	Mean dCq ± SD AAA (n = 83)	FC	*P* value	AUC (95% CI)
miR-145-5p	−1.2 ± 1.44	−2.0 ± 1.88	1.7	.0019^a^^,^^b^	0.65 (0.56-0.72)
miR-215-5p	−3.6 ± 1.93	−4.6 ± 2.36	1.9	.0034	0.63 (0.55-0.71)
miR-23a-3p	4.2 ± 1.36	3.6 ± 1.34	1.5	.0048^a^	0.63 (0.55-0.71)
miR-451a	6.1 ± 1.24	5.6 ± 1.74	1.5	.0077	0.62 (0.53-0.70)
miR-33a-5p	−3.7 ± 2.66	−4.7 ± 2.74	2.0	.0121^a^	0.62 (0.53-0.70)
miR-26a-5p	1.8 ± 1.04	1.5 ± 1.07	1.3	.0122	0.61 (0.53-0.70)
miR-93-5p	1.2 ± 1.41	0.7 ± 1.55	1.4	.0172	0.62 (0.53-0.70)
miR-24-3p	3.1 ± 1.61	2.6 ± 1.58	1.4	.0327	0.59 (0.51-0.68)
miR-192-5p	−1.9 ± 1.56	−2.4 ± 1.98	1.5	.0327	0.60 (0.51-0.68)
miR-125a-5p	−1.3 ± 1.47	−1.8 ± 1.86	1.4	.0394	0.58 (0.49-0.66)
miR-103a-3p	0.7 ± 1.30	0.3 ± 1.32	1.3	.0449	0.58 (0.50-0.66)

*AUC,* Area under curve; *CI,* confidence interval; *FC,* fold change; *SD,* standard deviation; *Cq,* quantitation cycle.

Table of the mean normalized Cq values with SD across the groups, and FC between the groups with the *P* value from Student *t* test.

a *P* < .05 when adjusted for age differences among groups.

b *P* < .05 in a multivariate forward conditional regression analysis including all significant miRs.

### Expression of miRs in plasma in patients with bilateral PA

Levels of seven miR (23a, 24, 93, 152, 215, 221, and 326) were altered (1.4-fold to 2.3-fold) in patients with bilateral PA compared with in those with unilateral PA (Table II). Only changes in miR-23a and miR-326 levels remained significant after adjusting for differences in age, which was the co-variate that differed between the groups. Only changes in miR-326 levels remained significant in a multiple regression analysis including all miRs.

**Table II:** MicroRNAs (*miRs*) in plasma that are different when comparing patients with unilateral and bilateral popliteal artery aneurysms (*PA*)

miR	Mean dCq ± SD unilateral PA (n = 78)	Mean dCq ± SD bilateral PA (n = 105)	FC	*P* value	AUC (95% CI)
miR-326	−4.5 ± 2.68	−5.7 ± 2.69	2.3	.0027^a^^,^^b^	0.62 (0.53-0.70)
miR-23a-3p	4.2 ± 1.50	3.7 ± 1.24	1.4	.0112^a^	0.62 (0.53-0.70)
miR-24-3p	3.2 ± 1.75	2.6 ± 1.46	1.5	.0162	0.61 (0.52-0.69)
miR-93-5p	1.2 ± 1.59	0.7 ± 1.39	1.4	.0221	0.61 (0.53-0.70)
miR-221-3p	1.8 ± 2.11	1.1 ± 2.00	1.7	.0233	0.59 (0.51-0.67)
miR-215-5p	−3.6 ± 2.05	−4.4 ± 2.23	1.6	.0281	0.61 (0.53-0.69)
miR-152-3p	−0.7 ± 1.61	−1.1 ± 1.36	1.4	.0449	0.59 (0.50-0.67)

*AUC,* Area under curve; *CI,* confidence interval; *FC,* fold change; *SD,* standard deviation; *Cq,* quantitation cycle.

The mean normalized Cq values with SD across the groups, and FC between the groups with *P* values from the Student *t* test.

a *P* < .05 when adjusted for age differences among groups.

b *P* < .05 in a multivariate forward conditional regression analysis including all significant miRs.

### Expression of miRs in plasma in patients with isolated PA and patients with multiple aneurysms

Four miR levels (93, 145, 215, and 326) were changed (1.4-fold to 1.9-fold) among patients with isolated PA compared with in those with multiple aneurysms (Table III). However, none of these changes remained after adjustment for age and hypertension or age, which were the covariates that differed between the groups. In a regression analysis, including all miRs, the change in miR-145 levels remained significant in patients with unilateral PA. The AUC for miR-145 combined with age and hypertension was slightly increased, from 0.62 to 0.69 (0.61-0.77). When comparing those with one or two aneurysms with those with more than two aneurysms, miR-451, -194, -125a, and -215 levels were significantly altered, with the change in miR-215 levels remaining significant in a multivariate model (data not shown). When comparing a group of 15 patients with 6 or 7 aneurysms to those with fewer than 6 aneurysms, levels of miR-215 and miR-451 were significantly altered. However, neither change remained significant in a multivariate model (data not shown). The miRNA concentration did not correlate with increasing number of aneurysms (data not shown).

**Table III:** MicroRNAs (*miRs*) in plasma that are different when comparing patients with or without isolated popliteal artery aneurysms (*PA*)

miR	Mean dCq ± SD isolated PA (n = 56)	Mean dCq ± SD multianeurysm (n = 127)	FC	*P* value^a^	AUC (95% CI)
miR-145	−1.1 ± 1.23	−1.8 ± 1.83	1.6	.0082^b^	0.62 (0.53-0.71)
miR-215-5p	−3.5 ± 1.90	−4.3 ± 2.26	1.7	.0281	0.61 (0.52-0.69)
miR-326	−4.5 ± 2.55	−5.5 ± 2.79	1.9	.0316	0.59 (0.5-0.68)
miR-93-5p	1.3 ± 1.39	0.8 ± 1.52	1.4	.0429	0.61 (0.52-0.69)

*AUC,* Area under curve; *CI,* confidence interval; *FC,* fold change; *SD,* standard deviation; *Cq,* quantitation cycle.

The mean normalized Cq values with SD across the groups, and FC between the groups with *P* values from the Student *t* test.

a No *P* values remained significant when adjusted for age and hypertension.

b *P* < .05 in a multivariate forward conditional regression analysis including all significant miRs.

### Correlations between miRs expressed in plasma among patients with PA

Most of the miRs correlated with each other to a certain degree (Table IV). However, miR-10b, 125a, 194, and 205 did not correlate strongly (r < 0.50) with other miRs (with some few exceptions), whereas miR-21, 23a, 24, 126, 152, and 335 correlated strongly (r > 0.80) with each other and moderately (r = 0.50-0.79) with several other miRs.

**Table IV:** Pearson's correlations between microRNAs (*miRs*) in the study population

miRs	10b	21	23a	24	26a	33a	93	99a	103a	125a	136	145	152	192	194	205	215	221	222	326	335	451	let7i
10b	–	0.42^a^^,^^b^	0.24^a^^,^^b^	0.20^a^^,^^b^	0.46^a^^,^^b^	0.26^a^^,^^b^	0.33^a^^,^^b^	0.42^a^^,^^b^	0.49^a^^,^^b^	0.33^a^^,^^b^	0.25^a^^,^^b^	0.19^a^^,^^c^	0.27^a^^,^^b^	0.18^a^^,^^c^	0.49^a^^,^^b^	0.37^a^^,^^b^	0.25^a^^,^^b^	0.24^a^^,^^b^	0.10^a^^,^^d^	0.22^a^^,^^b^	0.24^a^^,^^b^	0.46^a^^,^^b^	0.32^a^^,^^b^
21	0.42^a^^,^^b^	–	0.84^b^^,^^e^	0.83^b^^,^^e^	0.84^b^^,^^e^	0.69^b^^,^^f^	0.76^b^^,^^f^	0.49^a^^,^^b^	0.83^b^^,^^e^	0.42^a^^,^^b^	0.69^b^^,^^f^	0.47^a^^,^^b^	0.88^b^^,^^e^	0.27^a^^,^^b^	0.33^a^^,^^b^	0.24^a^^,^^b^	0.31^a^^,^^b^	0.87^b^^,^^e^	0.47^a^^,^^b^^,^^e^	0.70^a^^,^^b^^,^^f^	0.84^b^^,^^e^	0.47^a^^,^^b^	0.51^b^^,^^f^
23a	0.24^a^^,^^b^	0.84^b^^,^^e^	–	0.97^b^^,^^e^	0.82^b^^,^^e^	0.76^b^^,^^f^	0.82^b^^,^^e^	0.53^b^	0.78^b^^,^^f^	0.35^a^^,^^b^	0.65^b^^,^^f^	0.68^b^^,^^f^	0.82^b^^,^^e^	0.45^a^^,^^b^	0.24^a^^,^^b^	0.19^a^^,^^c^	0.45^a^^,^^b^	0.88^b^^,^^e^	0.56^b^	0.73^b^^,^^f^	0.86^b^^,^^e^	0.43^a^^,^^b^	0.53^b^^,^^f^
24	0.20^a^^,^^b^	0.83^b^^,^^e^	0.97^b^^,^^e^	–	0.80^b^^,^^e^	0.74^b^^,^^f^	0.84^b^^,^^e^	0.46^a^^,^^b^	0.77^b^^,^^f^	0.35^a^^,^^b^	0.64^b^^,^^f^	0.65^b^^,^^f^	0.84^b^^,^^e^	0.41^a^^,^^b^	0.23^a^^,^^b^	0.17^a^^,^^c^	0.43^a^^,^^b^	0.91^b^^,^^e^	0.56^b^	0.72^b^^,^^f^	0.87^b^^,^^e^	0.40^a^^,^^b^	0.49^b^^,^^f^
26a	0.46^a^^,^^b^	0.84^b^^,^^e^	0.82^b^^,^^e^	0.80^b^^,^^e^	–	0.63^b^^,^^f^	0.78^b^^,^^f^	0.55^b^	0.91^b^^,^^e^	0.50^b^^,^^f^	0.64^b^^,^^f^	0.57^b^^,^^f^	0.78^b^^,^^f^	0.41^a^^,^^b^	0.36^a^^,^^b^	0.30^a^^,^^b^	0.46^a^^,^^b^	0.79^b^^,^^f^	0.47^b^	0.60^b^^,^^f^	0.74^b^^,^^f^	0.50^b^^,^^f^	0.45^a^^,^^b^
33a	0.26^a^^,^^b^	0.69^b^^,^^f^	0.76^b^^,^^f^	0.74^b^^,^^f^	0.63^b^^,^^f^	–	0.57^b^^,^^f^	0.45^a^^,^^b^	0.61^b^^,^^f^	0.33^a^^,^^b^	0.61^b^^,^^f^	0.49^a^^,^^b^	0.68^b^^,^^f^	0.24^a^^,^^b^	0.12^d^	0.22^a^^,^^b^	0.24^a^^,^^b^	0.76^b^^,^^f^	0.39^b^	0.59^b^^,^^f^	0.70^b^^,^^f^	0.33^a^^,^^b^	0.42^a^^,^^b^
93	0.33^a^^,^^b^	0.76^b^^,^^f^	0.82^b^^,^^e^	0.84^b^^,^^e^	0.78^b^^,^^f^	0.57^b^^,^^f^	–	0.48^a^^,^^b^	0.82^b^^,^^e^	0.40^a^^,^^b^	0.54^b^^,^^f^	0.66^b^^,^^f^	0.73^b^^,^^f^	0.49^a^^,^^b^	0.42^a^^,^^b^	0.23^a^^,^^b^	0.54^b^^,^^f^	0.75^b^^,^^f^	0.52^b^	0.64^b^^,^^f^	0.70^b^^,^^f^	0.69^b^^,^^f^	0.57^b^^,^^f^
99a	0.42^a^^,^^b^	0.49^a^^,^^b^	0.53^b^^,^^f^	0.46^a^^,^^b^	0.55^b^^,^^f^	0.45^a^^,^^b^	0.48^a^^,^^b^	–	0.58^b^^,^^f^	0.32^a^^,^^b^	0.43^a^^,^^b^	0.52^b^^,^^f^	0.40^a^^,^^b^	0.43^a^^,^^b^	0.38^a^^,^^b^	0.20^a^^,^^b^	0.38^a^^,^^b^	0.39^a^^,^^b^	0.30^a^^,^^b^	0.40^a^^,^^b^	0.44^a^^,^^b^	0.49^a^^,^^b^	0.40^a^^,^^b^
103a	0.49^a^^,^^b^	0.83^b^^,^^e^	0.78^b^^,^^f^	0.77^b^^,^^f^	0.91^b^^,^^e^	0.61^b^^,^^f^	0.82^b^^,^^e^	0.58^b^	–	0.50^b^^,^^f^	0.66^b^^,^^f^	0.55^b^^,^^f^	0.77^b^^,^^f^	0.39^a^^,^^b^	0.37^a^^,^^b^	0.25^a^^,^^b^	0.43^a^^,^^b^	0.75^b^^,^^f^	0.46^a^^,^^b^	0.59^b^^,^^f^	0.74^b^^,^^f^	0.59^b^^,^^f^	0.53^b^^,^^f^
125a	0.33^a^^,^^b^	0.42^a^^,^^b^	0.35^a^^,^^b^	0.35^a^^,^^b^	0.50^b^^,^^f^	0.33^a^^,^^b^	0.40^a^^,^^b^	0.32^a^^,^^b^	0.50^b^^,^^f^	–	0.31^a^^,^^b^	0.34^a^^,^^b^	0.36^a^^,^^b^	0.20^a^^,^^b^	0.22^a^^,^^b^	0.33^a^^,^^b^	0.31^a^^,^^b^	0.39^a^^,^^b^	0.15^a^^,^^c^	0.28^b^	0.31^b^	0.40^a^^,^^b^	0.23^a^^,^^b^
136	0.25^a^^,^^b^	0.69^b^^,^^f^	0.65^b^^,^^f^	0.64^b^^,^^f^	0.64^b^^,^^f^	0.61^b^^,^^f^	0.54^b^^,^^f^	0.43^a^^,^^b^	0.66^b^^,^^f^	0.31^a^^,^^b^	–	0.38^a^^,^^b^	0.66^b^^,^^f^	0.19^a^^,^^b^	0.16^a^^,^^c^	0.19^a^^,^^c^	0.20^a^^,^^b^	0.67^bf^	0.32^a^^,^^b^	0.48^a^^,^^b^	0.65^b^^,^^f^	0.33^a^^,^^b^	0.35^a^^,^^b^
145	0.19^a^^,^^c^	0.47^a^^,^^b^	0.68^b^^,^^f^	0.65^b^^,^^f^	0.57^b^^,^^f^	0.49^a^^,^^b^	0.66^b^^,^^f^	0.52^b^	0.55^b^^,^^f^	0.34^a^^,^^b^	0.38^a^^,^^b^	–	0.48^a^^,^^b^	0.51^b^^,^^f^	0.25^a^^,^^b^	0.25^a^^,^^b^	0.45^a^^,^^b^	0.53^b^^,^^f^	0.40^a^^,^^b^	0.54^b^^,^^f^	0.49^a^^,^^b^	0.51^b^^,^^f^	0.54^b^^,^^f^
152	0.27^a^^,^^b^	0.88^b^^,^^e^	0.82^b^^,^^e^	0.84^b^^,^^e^	0.78^b^^,^^f^	0.68^b^^,^^f^	0.73^b^^,^^f^	0.40^a^^,^^b^	0.77^b^^,^^f^	0.36^a^^,^^b^	0.66^b^^,^^f^	0.48^a^^,^^b^	–	0.26^a^^,^^b^	0.22^a^^,^^b^	0.18^a^^,^^c^	0.27^a^^,^^b^	0.88^b^^,^^e^	0.40^a^^,^^b^	0.69^b^^,^^f^	0.86^b^^,^^e^	0.37^a^^,^^b^	0.41^a^^,^^b^
192	0.18^a^^,^^c^	0.27^a^^,^^b^	0.45^a^^,^^b^	0.41^a^^,^^b^	0.41^a^^,^^b^	0.24^a^^,^^b^	0.49^a^^,^^b^	0.43^a^^,^^b^	0.39^a^^,^^b^	0.20^a^^,^^b^	0.19^a^^,^^b^	0.51^b^^,^^f^	0.26^a^^,^^b^	–	0.42^a^^,^^b^	0.10^d^	0.68^b^^,^^f^	0.24^a^^,^^b^	0.43^a^^,^^b^	0.32^b^	0.27^a^^,^^b^	0.39^a^^,^^b^	0.54^b^^,^^f^
194	0.49^a^^,^^b^	0.33^a^^,^^b^	0.24^a^^,^^b^	0.23^a^^,^^b^	0.36^a^^,^^b^	0.12^d^	0.42^a^^,^^b^	0.38^a^^,^^b^	0.37^a^^,^^b^	0.22^a^^,^^b^	0.16^a^^,^^c^	0.25^a^^,^^b^	0.22^a^^,^^b^	0.42^a^^,^^b^	–	0.23^a^^,^^b^	0.49^a^^,^^b^	0.21^a^^,^^b^	0.16^a^^,^^c^	0.13^d^	0.17^a^^,^^c^	0.58^b^^,^^f^	0.27^a^^,^^b^
205	0.37^a^^,^^b^	0.24^a^^,^^b^	0.19^a^^,^^c^	0.17^a^^,^^c^	0.30^a^^,^^b^	0.22^a^^,^^b^	0.23^a^^,^^b^	0.20^a^^,^^b^	0.25^a^^,^^b^	0.33^a^^,^^b^	0.19^a^^,^^c^	0.25^a^^,^^b^	0.18^a^^,^^c^	0.10^d^	0.23^a^^,^^b^	–	0.12^d^	0.24^a^^,^^b^	0.06^d^	0.17^c^	0.15^a^^,^^c^	0.30^a^^,^^b^	0.14^d^
215	0.25^a^^,^^b^	0.31^a^^,^^b^	0.45^a^^,^^b^	0.43^a^^,^^b^	0.46^a^^,^^b^	0.24^a^^,^^b^	0.54^a^^,^^b^	0.38^a^^,^^b^	0.43^a^^,^^b^	0.31^a^^,^^b^	0.20^a^^,^^b^	0.45^a^^,^^b^	0.27^a^^,^^b^	0.68^b^^,^^f^	0.49^a^^,^^b^	0.12^d^	–	0.28^a^^,^^b^	0.47^a^^,^^b^	0.24^b^	0.27^a^^,^^b^	0.44^a^^,^^b^	0.47^a^^,^^b^
221	0.24^a^^,^^b^	0.87^b^^,^^e^	0.88^b^^,^^e^	0.91^b^^,^^e^	0.79^b^^,^^f^	0.76^b^^,^^f^	0.75^b^^,^^f^	0.39^a^^,^^b^	0.75^b^^,^^f^	0.39^a^^,^^b^	0.67^b^^,^^f^	0.53^b^^,^^f^	0.88^b^^,^^e^	0.24^a^^,^^b^	0.21^a^^,^^b^	0.24^a^^,^^b^	0.28^a^^,^^b^	–	0.46^a^^,^^b^	0.71^b^^,^^f^	0.88^b^^,^^e^	0.38^a^^,^^b^	0.40^a^^,^^b^
222	0.10^d^	0.47^a^^,^^b^	0.56^b^^,^^f^	0.56^b^^,^^f^	0.47^a^^,^^b^	0.39^a^^,^^b^	0.52^b^^,^^f^	0.30^a^^,^^b^	0.46^a^^,^^b^	0.15^a^^,^^c^	0.32^a^^,^^b^	0.40^b^	0.40^a^^,^^b^	0.43^a^^,^^b^	0.16^a^^,^^c^	0.06^d^	0.47^a^^,^^b^	0.46^a^^,^^b^	–	0.45^a^^,^^b^	0.44^a^^,^^b^	0.35^a^^,^^b^	0.53^b^^,^^f^
326	0.22^a^^,^^b^	0.70^b^^,^^f^	0.73^b^^,^^f^	0.72^b^^,^^f^	0.60^b^^,^^f^	0.59^b^^,^^f^	0.64^b^^,^^f^	0.40^a^^,^^b^	0.59^b^^,^^f^	0.28^a^^,^^b^	0.48^a^^,^^b^	0.54^b^^,^^f^	0.69^b^^,^^f^	0.32^a^^,^^b^	0.13^d^	0.17^a^^,^^c^	0.24^a^^,^^b^	0.71^b^^,^^f^	0.45^a^^,^^b^	–	0.70^b^^,^^f^	0.35^a^^,^^b^	0.57^b^^,^^f^
335	0.24^a^^,^^b^	0.84^b^^,^^e^	0.86^b^^,^^e^	0.87^b^^,^^e^	0.74^b^^,^^f^	0.70^b^^,^^f^	0.70^b^^,^^f^	0.44^a^^,^^b^	0.74^b^^,^^f^	0.31^a^^,^^b^	0.65^b^^,^^f^	0.49^b^	0.86^b^^,^^e^	0.27^a^^,^^b^	0.17^a^^,^^c^	0.15^a^^,^^c^	0.27^a^^,^^b^	0.88^b^^,^^e^	0.44^a^^,^^b^	0.70^b^^,^^f^	–	0.37^a^^,^^b^	0.48^a^^,^^b^
451	0.46^a^^,^^b^	0.47^a^^,^^b^	0.43^a^^,^^b^	0.40^a^^,^^b^	0.50^b^^,^^f^	0.33^a^^,^^b^	0.69^b^^,^^f^	0.49^a^^,^^b^	0.59^b^^,^^f^	0.40^a^^,^^b^	0.33^a^^,^^b^	0.51^b^^,^^f^	0.37^a^^,^^b^	0.39^a^^,^^b^	0.58^b^^,^^f^	0.30^a^^,^^b^	0.44^a^^,^^b^	0.38^a^^,^^b^	0.35^a^^,^^b^	0.35^a^^,^^b^	0.37^a^^,^^b^	–	0.55^b^^,^^f^
let7i	0.32^a^^,^^b^	0.51^b^^,^^f^	0.53^b^^,^^f^	0.49^a^^,^^b^	0.45^a^^,^^b^	0.42^a^^,^^b^	0.57^b^^,^^f^	0.40^a^^,^^b^	0.53^b^^,^^f^	0.23^a^^,^^b^	0.35^a^^,^^b^	0.54^b^^,^^f^	0.41^a^^,^^b^	0.54^b^^,^^f^	0.27^a^^,^^b^	0.14^d^	0.47^a^^,^^b^	0.40^a^^,^^b^	0.53^b^^,^^f^	0.57^b^^,^^f^	0.48^a^^,^^b^	0.55^b^^,^^f^	–

a Significant correlations of <0.50.

b Correlation is significant at the .01 level.

c Correlation is significant at the .05 level.

d No statistical significant correlations.

e Correlations with r-values between 80 and 99.

f Correlations with r-values between 50 and 79.

The miR-93 and -215 levels differed between all groups compared, miR-24 and -23a levels were altered in bilateral PA and AAA, miR-145 levels were altered in isolated PA and AAA, and miR-326 levels were altered in isolated or bilateral PA. The Fig shows miRs for which alterations overlapped in the different comparisons. Supplementary Table IV shows the 14 different miRs that were altered in the different subgroup analysis and the potential genes they target as determined by in silico analysis using the miRWalk database.

**Fig:**
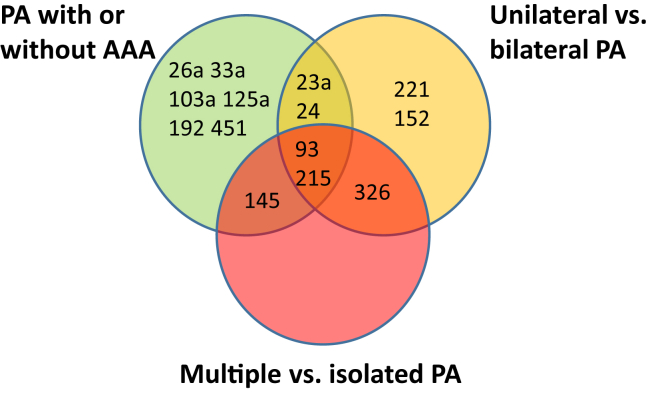
Overlapping significant changed microRNAs (miRs) in patients with popliteal artery aneurysm (*PA*) with or without abdominal aortic aneurysm (*AAA*), bilateral PA or multiple aneurysms.

## Discussion

Gene regulation is fine-tuned by miRs, which are implicated in numerous diseases. We screened patients with PA for candidate miRs previously investigated in the aneurysm field and for relevant targets within vascular biology.

A study by Busch and colleagues^12^ investigated the expression of miRs in biopsies from 8 PA and 19 AAA aortas. The expression pattern tended to differ between the groups. Compared with controls, there was a downregulation of miR-362, miR-19b miR-1, miR-194, miR-769, miR-21, and miR-550 in AAA and downregulation of miR-19b-1 and miR-769 in PA. MiR-362 and miR-194 showed no change and miR-550 and miR-21 were upregulated in PA. In this study, which included the analysis of miR-21 and miR-194 in plasma from patients with PA or concomitant AAA, these miRs were not changed significantly. A simple explanation for this discrepancy is that plasma levels do not always reflect tissue levels and vice versa, or depends on the repair of the PA in our study.

When comparing data on patients with PA with our previously published article on AAA patients,^7^ we can observe that eight of the 14 miRs were changed in different ways in the subgroups of patients with PA. Six of the 14 miRs (miR-33a, miR-103a, miR-125a, miR-192, miR-215, and miR-451) were altered in patients with PA with concomitant AAA. Among the miRs, miR-103a has previously been identified among AAA patients in a study by Plana et al,^13^ as being downregulated 1.4-fold in plasma of AAA patients when unadjusted for covariates and upregulated 1.3-fold in aortic tissue from AAA patients. This miR was slightly upregulated in plasma from AAA patients in our previous study. Interestingly, in light of our results, Nakao et al^14^ recently found that genetic ablation of miR-33 in two separate aneurysm models attenuated AAA formation via decreased macrophage accumulation. Our data indicate that an underlying PA potentiates the secretion of miRs compared with when having only an AAA or that alteration in the secretion of miRs affects the clinical outcome of developing AAA or PA. The six miRs that were previously studied by us in AAA patients and were not changed among patients with PA were miR-10b, miR-99a, miR-136, miR-194, miR-205, and miR-let-7i.

In our analysis, we also included miRs that have been associated with aneurysmal disease or vascular biology in the literature and found that some of these were altered among the patients with PA, namely, miR-23a,^15^ miR-24,^16^ miR-26a,^17^ miR-93,^18^, ^19^, ^20^ miR-145,^21^ and miR-152.^22^^,^^23^ In our study, miR-23a was upregulated by 50% in plasma from patients with PA with concomitant AAA or bilateral PA, which is similar to what was observed in plasma in patients with aortic dissection,^15^ although dissection should be considered a specific entity. Two miRs miR-93 and miR-215—differed in all of the compared subgroup analysis (Fig). MiR-93 showed a similar expression as miR-23 in our study, although miR-93 also showed higher expression in plasma from patients with multiple aneurysms. MiR-93 has not previously been associated with aneurysmal disease, to our knowledge. However, studies have shown that miR-93 may regulate vascular endothelial growth factor in hyperglycemic conditions and in mononuclear cells in Kawasaki disease.^19^^,^^20^ In a study by Sullivan et al,^18^ plasma miR-93 was identified as an independent predictor of the presence of coronary artery disease and Feng et al^24^ showed that miR-93 regulate smooth muscle cell proliferation, which is an important feature in aneurysm disease. MiR-215 is altered in atherosclerotic plaque, decreases endothelial cell recruitment, increases endothelial cell apoptosis, and stimulates a switch from contractile smooth muscle cell phenotype to a synthetic form, all hallmarks in aneurysm disease.^25^ The fact that there are miRs that are commonly altered in different subgroups and others that are specific in some subgroups indicates that there are different pathophysiologic pathways involved in the different aneurysms. Further, miR-26a was downregulated in mouse models of aneurysm formation in a previous study.^17^ The role of miR-145 was elegantly elucidated by Wu et al^21^ by overexpressing miR-145 through lentivirus infection, which decreased the incidence of AAA. MiR-145 was the most significantly altered miR among those investigated in our study, being upregulated in the plasma of patients with PA with concomitant AAA or multiple aneurysms. MiR-152 has not been associated with aneurysm previously. One previous study has shown that miR-152 is expressed in atherosclerotic plaques in macrophages^22^ and another study showed that miR-152 is involved in inhibiting apoptosis in microvascular endothelial cells in the human brain.^23^

One of the most altered miRs in this study was miR-326, which had a 2.3-fold higher expression among patients with bilateral PA. This miR is relatively unstudied in the vascular biology field, apart from in our previous study,^7^ where it was one of the most altered miRs. Most research regarding miR-326 has thus far been focused on various cancers and graft-versus-host disease, but is an interesting target when investigating the pathophysiology of aneurysmal disease.

An important limitation of this study is that we have only measured miRs in the peripheral circulation, not in the tissues of the different aneurysms. We did not study and cannot state whether circulated or locally produced miRs affect the clinical outcome of AAA or PA disease or if the secretion of miR is a consequence of the disease. The overall technical development toward less invasive endovascular surgery, and the fact that biopsies of the arteries cannot be performed safely, creates a great challenge when it comes to investigating tissue samples of aneurysmal disease, however*.* Furthermore, some of the aneurysms had been operated on at the time when blood sampling took place. The fact that an aneurysm is repaired surgically does not mean that the underlying disease is cured, however. When an aneurysm is repaired, it is not removed, but typically bypassed or treated with a stent graft, leaving most of the diseased tissue in the patient, although with less contact with the blood flow. It is also common that the nonrepaired arteries are wider compared with healthy individuals.

Another limitation of this study is the lack of a control group, without aneurysmal disease. We have compared patients with AAA and aneurysm healthy controls in a previous investigation,^7^ but unfortunately the analytic method was changed making direct comparisons impossible.

## Conclusions

The results from this study indicate that the miR signature in patients with PA shares some similarities with that of AAA, but also has some distinct patterns. The identified miRs and the genes they regulate could be potential targets for further studies to increase the understanding of aneurysmal pathogenesis in general.

## Author contributions

Conception and design: DW, HR, AW, HI, MB

Analysis and interpretation: DW, AW, MB

Data collection: DW, HR, AW, HI, MB

Writing the article: DW, AW, MB

Critical revision of the article: DW, HR, AW, HI, MB

Final approval of the article: DW, HR, AW, HI, MB

Statistical analysis: DW

Obtained funding: DW, AW, HI, MB

Overall responsibility: DW

## Appendix

Supplementary Table I

Supplementary Table II

Supplementary Table III

Supplementary Table IV
