# Supplementary material for: Circulating microRNA in patients with popliteal and multiple artery aneurysms
Source: JVS Vasc Sci. 2021 May 15;2:129–35. doi: 10.1016/j.jvssci.2021.04.003 (PMC8489194; doi:10.1016/j.jvssci.2021.04.003)
Supplement: Supplementary Table I [file mmc1.docx]

**Supplemental table 1**

**Patient characteristics among PA patients with or without concomitant AAA**

|  | No AAA | AAA | P-value |
| --- | --- | --- | --- |
| No. of patients | 100 | 83 |  |
| Median age (years) | 68.0 | 74.0 | <0.001 |
| Gender (M/F) | 94.0% | 98.8% | 0.093 |
| Active smokers | 23.0% | 20.5% | 0.72 |
| Ever smokers | 73.0% | 85.5% | 0.047 |
| Hypertension (%) | 61.6% | 74.4% | 0.08 |
| Family history (%) | 70.7% | 62.7% | 0.27 |

AAA: abdominal aortic aneurysm, PA: popliteal artery aneurysm.
