# Supplementary material for: Circulating microRNA in patients with popliteal and multiple artery aneurysms
Source: JVS Vasc Sci. 2021 May 15;2:129–35. doi: 10.1016/j.jvssci.2021.04.003 (PMC8489194; doi:10.1016/j.jvssci.2021.04.003)
Supplement: Supplementary Table II [file mmc2.docx]

**Supplemental table 2**

**Patient characteristics among PA patients with unilateral and bilateral aneurysms**

|  | unilateral PA | bilateral PA | P-value |
| --- | --- | --- | --- |
| No. of patients | 78 | 105 |  |
| Median age (years) | 68.0 | 73.0 | 0.018 |
| Gender (M/F) | 93.6% | 98.1% | 0.12 |
| Active smokers | 23.1% | 21.0% | 0.86 |
| Ever smokers | 78.2% | 79.0% | 1.00 |
| Hypertension (%) | 60.3% | 72.8% | 0.081 |
| Family history (%) | 35.9% | 30.8% | 0.53 |

PA: popliteal artery aneurysm
