# Supplementary material for: Circulating microRNA in patients with popliteal and multiple artery aneurysms
Source: JVS Vasc Sci. 2021 May 15;2:129–35. doi: 10.1016/j.jvssci.2021.04.003 (PMC8489194; doi:10.1016/j.jvssci.2021.04.003)
Supplement: Supplementary Table III [file mmc3.docx]

**Supplemental table 3**

**Patient characteristics among PA patients with an isolated aneurysm and those with multiple aneurysm disease**

|  | Isolated PA | Multiple aneurysm | P-value |
| --- | --- | --- | --- |
| No. of patients | 56 | 127 |  |
| Mean no. of aneurysms | 1 | 2.9 |  |
| Median age (years) | 67.0 | 72.0 | 0.002 |
| Gender (M/F) | 92.9% | 97.6% | 0.12 |
| Active smokers | 21.4% | 22.0% | 1.00 |
| Ever smokers | 71.4% | 81.9% | 0.12 |
| Hypertension (%) | 53.6% | 73.6% | 0.01 |
| Family history (%) | 33.9% | 32.5% | 0.87 |

PA: popliteal artery aneurysm.
